# Y genetic variation and phenotypic diversity in health and disease

**DOI:** 10.1186/s13293-015-0024-z

**Published:** 2015-03-13

**Authors:** Laure K Case, Cory Teuscher

**Affiliations:** Department of Medicine, University of Vermont, 89 Beaumont Ave, Burlington, VT 05405 USA; Department of Pathology, University of Vermont, 89 Beaumont Ave, Burlington, VT 05405 USA; University of Vermont, Given Medical Building C317, Burlington, VT 05405 USA

**Keywords:** Y chromosome, Cardiovascular, Immune system, Cancer, Copy number variation, Gene regulation

## Abstract

Sexually dimorphic traits arise through the combined effects of sex hormones and sex chromosomes on sex-biased gene expression, and experimental mouse models have been instrumental in determining their relative contribution in modulating sex differences. A role for the Y chromosome (ChrY) in mediating sex differences outside of development and reproduction has historically been overlooked due to its unusual genetic composition and the predominant testes-specific expression of ChrY-encoded genes. However, ample evidence now exists supporting ChrY as a mediator of other physiological traits in males, and genetic variation in ChrY has been linked to several diseases, including heart disease, cancer, and autoimmune diseases in experimental animal models, as well as humans. The genetic and molecular mechanisms by which ChrY modulates phenotypic variation in males remain unknown but may be a function of copy number variation between homologous X-Y multicopy genes driving differential gene expression. Here, we review the literature identifying an association between ChrY polymorphism and phenotypic variation and present the current evidence depicting the mammalian ChrY as a member of the regulatory genome in males and as a factor influencing paternal parent-of-origin effects in female offspring.

## Review

### Introduction

Like autosomal chromosomes (Chrs), the sex chromosomes (ChrX; ChrY) are thought to have once been identical pairs that were free to recombine and exchange genetic material. Over the course of evolution, ChrY became unique from all other Chrs with the acquisition of a dominant sex-determining gene and subsequent chromosomal inversions that restricted recombination with its homologous ChrX, that led to its degradation [1,2]. The relatively few protein-coding genes on ChrY are predominantly male-specific genes acquired through transposition and translocation from other Chrs [3,4]. The remainder of ChrY is largely ampliconic containing protein-coding and non-protein-coding sequences. These features of ChrY have led to the consensus that it is primarily composed of “junk” DNA whose contribution to phenotypic differences among the sexes is limited to sexual development and spermatogenesis. Indeed, ChrY is not essential to life, and large deletions in the long arm of the murine ChrY have no overt phenotypic effects except on spermatogenesis and sex ratio distortion [5-7]. Nonetheless, a rapidly growing body of research has identified an association between the mammalian ChrY and biological functions not directly related to male reproduction.

Animal models have been useful for investigating the effects of sex Chrs on complex traits. Two of the most referenced models in this review are ChrY consomic mice and the “four core genotypes” (FCG) mouse model, both of which have been recently reviewed in detail [8]. ChrY consomic strains provide insight into the effects of natural genetic variation in ChrY on male phenotypes. They are generated by intercrossing a male mouse with a given ChrY to a female mouse with the desired genetic background, such as C57BL/6 J (B6). After a series of backcrosses of 10 generations or more to B6 females, the autosomes, ChrX, the peudoautosomal regions of ChrX and ChrY, and the mitochondrial genome of the strain donating ChrY are replaced with the B6 genome. Therefore, the genetic variation among ChrY consomic strains derives from the non-recombining region of ChrY called the nonpseudoautosomal or male-specific region of ChrY (MSY) [9].

The MSY is composed of a long arm and a short arm. The long arm of the MSY is comprised of an ampliconic sequence repeating about 200 times that contains genes critical for spermatogenesis, which are amplified to varying degrees among different *Mus* species [10,11]. The short arm encodes 12 families of protein-coding genes [10], including the testes-determining gene *Sry* (sex-determining gene of ChrY). The existence of functionally significant *Sry* polymorphisms is well documented in studies using B6-ChrY consomic strains, where *Sry* alleles give rise to varying degrees of sex-reversal, ranging from normal testis development to permanent sex-reversal [12-17]. Therefore, *Sry* polymorphisms could lead to differences in neonatal and/or adult testosterone levels among B6-ChrY consomic strains. Studying multiple B6-ChrY consomic strains for concordance between the strain distribution patterns of *Sry* polymorphisms with the phenotype of interest can shed light on whether hormones may be influencing the trait.

The FCG mouse model was designed to investigate the contribution of sex Chrs (XX vs. XY) or gonadal type (ovaries vs. testes) on disease. It has been useful in uncoupling sex hormone effects from sex Chr effects. This mouse model was made possible due to two genetic mutations. First, a genetic mutation in the retrovirally infected EK.CCE embryonic stem cell line (from 129/SvEv mice) [18,19] deleted the region containing the testes-determining gene *Sry* on ChrY, resulting in the development of XY female mice [20]. Second, transgenic mice were constructed with autosomal expression of *Sry*, which complements the *Sry* deletion transmitted by female founder mice derived from the ESC line [21]. In the FCG model, when XX and XY mice with the same gonadal type differ in phenotype, the difference is attributed to sex Chr complement (the number of ChrX or the presence of ChrY).

In this review, we discuss the growing body of research exploring the ability of the mammalian ChrY to regulate physiology and disease in males. We focus on those areas of biology with which ChrY is not historically associated and refer the reader to an informative review [22], together with the more recent references in [23] for ChrY’s role in male reproduction and spermatogenesis, or brain and behavior phenotypes [24,25]. Finally, we highlight current evidence for the evolutionary conservation of ChrY as a member of the regulatory genome in males due to its ability to modulate genome-wide gene expression.

#### Growth and metabolism

Pre-gonadal sexual dimorphisms provide evidence for phenotypic differences being driven by sex Chrs independently of the actions of sex hormones. In mammals, differences in developmental rate precede the production of gonadal hormones, with XY individuals showing increased growth compared to XX individuals [26]. For example, an increase in cell number among XY preimplantation embryos [27] is due to ChrY [28]. Furthermore, XY fetuses are larger because they are more developmentally advanced than XX fetuses in the same litter, and the contribution of ChrY to this difference varies with the strain origin of ChrY [29]. Genetic differences in ChrY also regulate cardiomyocyte size and cardiac mass [30]. In reciprocal ChrY consomic strains between B6 and A/J mice, B6-ChrY^A/J^ and A/J-ChrY^B6^ mice have smaller and larger cardiomyocyte sizes than wild-type B6 and A/J mice, respectively [30]. The reduction in cardiomyocyte size is due to the absence of the hypertrophic effects of post-pubertal testosterone on these cells and is a direct consequence of ChrY-encoded polymorphisms [31].

Adult body weight may also be under the control of ChrY. Using of a panel of 17 ChrY consomic mouse strains on the DH/Sgn background, a continuous distribution in body weight in adult mice was identified [32]. The effect of ChrY from KK/Ta on body weight was independent of the autosomal and ChrX genetic background, thus supporting the interpretation that ChrY contains genes that control body size in mice [32]. In the FCG model on the MF1 genetic background, it was observed that the presence of two sex Chrs, either XX or XY, increases body weight and adiposity compared to mice with only one ChrX (X0 mice) [33]. The similarity between mice with either XX or XY suggests that both sex Chrs possess genetic elements that influence metabolism independently of gonadal hormones and that these effects may arise from the presence of the XY paralogs [33].

In humans, ChrY-mediated differences in growth are observed as adult height differences between the sexes. This ChrY effect was first observed in cases in which abnormal stature was the result of XX or XY gonadal dysgenesis, in which people have gonadal steroid deficiency irrespective of sex Chr complement. Therefore, similar to the FCG mouse model, phenotypic differences between XY and XX individuals with gonadal dysgenesis can be directly linked to differences in sex Chrs, rather than gonadal sex hormones [34]. The mean adult height of people with XY is significantly higher than in people with XX gonadal dysgenesis, leading to the conclusion that ChrY itself has genetic elements that promote growth independently of gonadal sex steroids [35]. Furthermore, data suggest that the 0.5- to 2-cm difference in height reported between pre-pubescent boys and girls [36] may be driven by ChrY, thus contributing early to the large difference in height manifested after the production of sex hormones in adulthood [37].

#### Cardiovascular diseases

A male-biased sexual dimorphism exists in the incidence and prevalence of many cardiovascular diseases [38]. Blood pressure is a risk factor associated with cardiovascular disease, and rodent models of hypertension have identified ChrY as a contributor to hypertension in males. A rat model was used to investigate the genetic mechanism driving spontaneous hypertension in SHR (S) rats compared to the normotensive Wistar-Kyoto (W) rat [39]. Male offspring from the W × S cross have higher blood pressure than males from the S × W cross, with no effect on females in either cross. Blood pressure in offspring of reciprocal F_2_ intercrosses was consistent with the presence of a locus on ChrY, suggesting that hypertension is mediated by ChrY. In an experimental mouse model of hypertension using FCG mice, gonad-intact XY males exhibit increased blood pressure compared to XX females. In contrast, gonadectomized (GDX) XX mice had greater mean arterial pressure compared to GDX XY mice, regardless of gonadal sex [40]. This raises the possibility that the contribution of hormones and sex Chrs to hypertension are in opposition to each other, generating effects that reduce the overall sex differences driven by one or the other.

Genetic variation in the human ChrY contributes significantly to the quantitative variation of male diastolic blood pressure in the overall population [41]. Polish and Scottish men inheriting a ChrY with one (*HindIII*^+^) of two biallelic markers had a significantly higher systolic and diastolic pressure [42], but this association is not consistent across human studies. Research on cardiovascular risk factors, including blood pressure, cholesterol levels, and body mass index, among Polish and Japanese men failed to identify a significant ChrY association [43,44]. The inconsistency between ChrY haplotype and hypertension in men led to a study examining the effects of ChrY on blood pressure in pre-pubertal boys [45]. They observed that blood pressure was higher in *HindIII*^−^ boys in the period before, and after, pubertal growth. These boys were also younger at the onset of peak height growth, suggesting that genetic variation in ChrY may influence blood pressure and height in a sex hormone-independent fashion.

Coronary artery disease is a male-biased cardiovascular disease that is strongly associated with genetic variation in ChrY. A study on a cohort of British men identified a 50% higher risk of coronary artery disease in men inheriting ChrY haplogroup I compared to other ChrY haplogroups [46]. This association was independent of any traditional cardiovascular risk factors, including blood pressure, lipids, glucose, body mass index, C-reactive protein, creatinine, and insulin resistance [46,47]. The characterization of the macrophage transcriptome between haplogroup I *vs.* other ChrY haplogroups identified differential expression in genes related to inflammation and immunity [46]. Furthermore, macrophages from haplogroup I showed downregulation in the expression of the ChrY genes *UTY* and *PRKY* [47], suggesting that the ChrY-mediated influence on coronary artery disease may be linked to differential expression of ChrY genes on the immune system.

#### Immune system, infectious diseases, and autoimmunity

Sex-specific differences exist in many aspects of immune system physiology and contribute to the pathogenic differences in autoimmune and infectious disease observed between males and females [48]. The female immune response against many infectious pathogens tends to be more robust, leading to a better prognosis in disease outcome. However, the evolutionary advantage of this heightened female immune response also contributes to their higher risk of developing autoimmune disease. While these sex differences in immunity are predominantly linked to the differential effects of sex hormones on immune cells, ChrY can also influence the immune response and susceptibility to disease [46,49-53].

Mutations in the murine ChrY are also associated with deficiencies in B cell, NK cell, and iNKT cell development, suggesting a role for ChrY in the proper development of the immune system. B and NK cell deficiencies and Peyer’s patch defects were observed among a novel immunodeficient mouse strain arising from a spontaneous ChrY mutation on the C57BL/6 N background. B and NK cell populations gradually diminished from 3 weeks of age in male mice and were completely absent by 10 weeks of age [54]. An analysis of ChrY revealed that it was one third shorter than expected, and exome sequencing did not identify any additional mutations. Thus, these findings were associated with a ChrY long arm deletion, which adds to the accumulating evidence (discussed later in the review) for gene regulatory roles by genetic information on the long arm of mouse ChrY.

Male, but not female, mice genetically deficient for IFN-αβR1 lack Vα14 + iNKT cells, yet conventional T and NK cells remained unaffected [55]. This deficiency is linked to ChrY and is independent of IFN-αβ. Female bone marrow was capable of reconstituting all lymphocyte compartments and, in the context of lymphopenia-induced proliferation, female IFNαβR1^−/−^ thymocytes, including Vα14 + iNKT cells, survived and proliferated in both male and female hosts. These findings demonstrate that sex hormones do not cause the loss of iNKT cells in male mice and that the iNKT defect is cell autonomous and driven by ChrY [55]. Furthermore, iNKT cell number among a panel of B6-ChrY consomic strains shows a continuous distribution in the percentage of basal iNKT cells among male mice, suggesting that natural genetic variation in ChrY influences the development of these cells [52]. However, the ChrY from IFNαβR1^−/−^ male mice, which was transmitted by male founder mice derived from 129/SvEv embryonic stem cells, is unique, as male mice inheriting the wild-type 129/SvEv ChrY by natural breeding do not exhibit the deficiency in iNKT cell numbers. This strongly suggests the presence of a unique translocation or deletion in the ChrY of IFNαβR1^−/−^ male mice. Interestingly, male founders transmitting both the decrease in iNKT cells and the *Sry* deletion were derived from the same 129/SvEv ESC line, suggesting that phenotypic variation associated with ChrY transmitted by male founders from this line may reflect non-naturally selected ChrY genetic variation [56,57].

There is a documented association between ChrY and susceptibility to infectious diseases in mice and humans. We have shown that natural genetic variation in ChrY influences the survival rate of male B6-ChrY consomic mice infected with coxsackievirus B3 (CVB3) [52]. Furthermore, using the FCG model, we showed that compared to GDX XX mice, GDX XY mice exhibited less severe CVB3-induced myocarditis, an inflammatory heart disease that predominates in both men and male experimental mice [58,59], indicating that myocarditis susceptibility is influenced by ChrX and/or ChrY [51]. Then, using B6-ChrY consomic mice, we found that myocarditis severity was influence by natural genetic variation in ChrY and specifically associated with copy number variation in ChrY multicopy genes [60]. In humans, an association has been made between ChrY haplogroup and AIDS progression in HIV-infected men. Among European Americans, men inheriting ChrY haplogroup I show accelerated progression to AIDS and related death, as well as delayed HIV-1 viral suppression during HAART therapy, compared to other ChrY haplogroups [61]. A genetic evaluation of ChrY haplogroup I has not identified the particular genetic variant associated with AIDS progression [62].

Animal models of multiple sclerosis, including experimental allergic encephalomyelitis (EAE) and the cuprizone-induced demyelination model, have been widely used to explore the effects of ChrY on the sexual dimorphism in disease susceptibility. The observed effects have been highly dependent on the strain of mice used, the method employed to study sex Chr effects, and the disease model. In the FCG model, GDX XX SJL mice exhibited greater severity in EAE compared to GDX XY SJL mice, regardless of their gonadal type, suggesting that increased EAE in XX mice is due to the XX vs. XY sex chromosome complement difference, either through an XX EAE promoting effect relative to a single X or through an inhibitory effect of ChrY compared to having two ChrX [63]. Subsequent studies in the ChrY consomic model extended the findings from the FCG model by pointing to an inhibitory effect by the SJL ChrY on EAE susceptibility relative to the other SJL ChrY consomic strain studied [50]. Furthermore, the generation of SJL FCG bone marrow chimeras identified an effect of XY on CNS neurodegeneration, where having an XY CNS confers greater spinal cord and cerebellar pathology in SJL chimeric mice reconstituted with the same immune system, but differing in the sex Chr complement in the CNS [64]. When EAE was studied in the B6 FCG model, no difference in disease severity was observed between the genotype combinations [63]. In contrast, B6-ChrY consomic stains of mice identified a robust difference in EAE severity driven by polymorphic differences in ChrY and revealed varying degrees of sexual dimorphism in EAE severity across the consomic strains when compared to B6 female mice [60]. In the cuprizone CNS demyelination model, inherent sex differences exist in remyelination that persist after GDX of adult mice. B6 GDX male mice remyelinate to a lesser extent compared to GDX female mice [65], in that the rate of remyelination and the number of proliferating oligodendrocytes is decreased in FCG B6 XY vs. XX mice of both gonadal sexes [66].

#### ChrY aberrations in cancer

The vast majority of cancers have higher incidence and age-adjusted mortality rates in males compared to females [67,68]. ChrY abnormalities, including loss or gain of the entire Chr, long arm deletions, and transcriptional deregulation of ChrY genes, have been reported for numerous cancers [69]. In humans, the loss of ChrY from peripheral blood mononuclear cells (PBMC) is associated with risk of all-cause mortality and risk of cancer not driven by the hematopoietic system, with the median survival time reduced by 5.5 years among men with ChrY loss in PBMC [70]. The ChrY anomalies primarily occur within the cancer cells of the individual but may also appear in non-tumor cells at a low frequency, suggesting that ChrY abnormalities in cancer occur through a post-zygotic mechanism. Importantly, although the loss of ChrY from bone marrow-derived cells is considered to be an age-related event, an age-independent loss of ChrY is also a tumor associated abnormality.

The loss of ChrY has been frequently observed in prostate cancer, which is among the leading causes of cancer deaths among American men [71]. An experimental mouse model was generated to study the effects of ChrY on the tumorigenicity of the human prostate cancer cell line PC-3, which lacks ChrY. When a human ChrY was incorporated into this cell line, tumor suppression was observed in nearly all the athymic nude mice studied [72]. A bacterial artificial Chr microarray, containing clones from human ChrY, was used to identify a common deletion in Yp11.2 in prostate tumors [73] that contains about 30–60 copies of the *TSPY* gene. *TSPY* expression is restricted to testicular tissue and its primary physiological function remains ill defined, but it may participate in germ cell proliferation and meiosis [74]. Copy number variation (CNV) in *TSPY* in PBMC was also associated with the incidence of prostate cancer [73]. The dysregulation of *TSPY* expression has been linked to a number of other cancers [75], suggesting that CNV and the expression of *TSPY* may contribute to tumor progression in men. Thus, while the role of ChrY in cancer formation and progression remains unclear, ChrY genes or elements may be important for preventing the transformation of cells.

#### Regulation of autosomal gene expression by ChrY in males

Studies in *Drosophila* have identified global gene regulatory properties by ChrY, which has recently been reviewed in detail elsewhere [76]. There is also emerging evidence that the mammalian ChrY is a member of the regulatory genome in males and directly influences autosomal gene expression and thereby may play an important role in male physiology and disease states. In a mouse model of atherosclerosis, which exhibits a male-biased sexual dimorphism, the combined analysis of quantitative trait loci (QTL) mapping with gene expression profiling (eQTL) of bone marrow-derived macrophages from (AKR × DBA) F_2_ cohort identified a strong sex bias in gene expression [77]. Remarkably, >30% of the differentially expressed genes exhibited a male or female expression bias. Furthermore, whereas the majority of cis-eQTLs were shared between males and females, trans-eQTLs were primarily sex-specific. In males, ChrY represented a hotspot for trans-eQTLs, with 334 ChrY eQTLs identified. Unlike QTL mapping of autosomes and ChrX, it is not possible to associate a narrow linkage peak with a particular trait, since the majority of ChrY does not undergo recombination, thus restricting the linkage unit to the entire non-PAR region of ChrY. Nonetheless, this study led to the characterization of ChrY as a global regulator of genome-wide gene expression in mice [77].

Using B6-ChrY consomic strains of mice, we demonstrated the magnitude of ChrY’s ability to act as a trans-eQTL and epigenetically regulate the transcriptome, particularly in relation to the expression of alternatively spliced isoforms, in CD4^+^ T cells [60]. We also found that ChrY exerts cell-type-specific effects on gene regulation depending on the autosomal background of the mice. ChrY had a greater influence on the transcriptome of macrophages compared to CD4^+^ T cells in SJL-ChrY consomic mice, in which the sexual dimorphism in EAE is primarily due to macrophage function [78-80]. An analysis of CNV in ChrY multicopy genes identified an inverse correlation between copy number and the upregulation of genome-wide gene expression enriched for chromatin remodeling genes, thus providing a link between CNV and the ChrY trans-eQTL regulatory properties [60]. The multicopy genes associated with the regulatory properties of ChrY, including *Sly*, *Ssty1*, *Ssty2*, and *Rbmy* [11], have testis-specific expression patterns, thus contributing to the possibility that CNV in these genes may have epigenetic consequences in tissues where they are not expressed, such as immune cells. ChrY polymorphism also underlies differential gene expression in cardiomyocytes and mediates the hypertrophic response of these cells to post-pubertal testosterone [31]. This response was further shown to be mediated by an effect of ChrY polymorphism on the differential distribution of androgen receptors in the heart, accompanied by differences in chromatin architecture [81], suggesting the genetic differences in ChrY can mediate chromatin dynamics in this organ [31].

In human cells, ChrY simple sequence repeats have enhancer blocking activity [82], demonstrating a regulatory role for ChrY in coordinated gene expression [83]. Furthermore, the link between susceptibility to coronary artery disease and ChrY haplogroup I occurs in parallel with distinct autosomal and ChrX transcriptional profiles in macrophages [46], indicating that ChrY may influence susceptibility to coronary artery disease by regulating the gene expression profiles of pathogenic immune cells in men.

#### Transgenerational traits and paternal parent-of-origin (POO) effects linked to ChrY

The inheritance of transgenerational changes in phenotypes allows for organisms to rapidly transmit epigenetic information to their offspring and is postulated to be a factor accounting for the missing heritability underlying susceptibility to complex diseases [84]. The majority of evidence for transgenerational epigenetic inheritance involves environmentally induced phenotypes that are maternally transmitted to offspring [85]. Several studies have also documented transgenerational environmental effects arising from epigenetic modifications in male germ cells [86-90]. Interestingly, evidence for paternal transgenerational genetic effects on the transmission of phenotypes to female offspring in the absence of altered environmental stimuli has been documented and linked to ChrY [84]. Using consomic strains of mice while controlling for effects of social and environmental factors, this study found that transgenerational effects on various physiological and behavioral traits are common among female offspring of sires with different ChrYs.

X-Y intragenomic conflict has been proposed as a mechanism that may influence the structure of the sperm epigenome and contribute to fetal development [91]. In mice, CNV between the *Slx/Slxl1/Sly* [92,93] and *Sstx/Ssty1* [94] multicopy gene homologs contributes to X-Y intragenomic conflict. X-Y intragenomic conflict causes sex-ratio distortion in favor of the sex chromosome harboring more copies of the distorting gene [92,93,95]. In addition, X-Y intragenomic conflict leads to sperm head defects [6,7,96-99], as well as altered chromatin remodeling and sex chromosome gene expression in developing spermatids [91,100-102]. In B6-ChrY consomic strains of mice, we used the current information on the predicted number of B6 ChrY multicopy gene families with ChrX homologs, including *Sly*, *Rbmy*, *Ssty1*, *Ssty2*, *Rbm31y*, *and Srsy* [10], to investigate the extent of CNV in these multicopy ChrY genes among the B6-ChrY consomic panel of mice [11]. Phenotypes associated with CNV between X-Y homologs, including sex-ratio distortion and sperm head abnormalities, were identified in B6-ChrY consomic strains of mice inheriting a *M. m. domesticus* ChrY. Multiple regression analyses between each of these phenotypes and ChrY multicopy gene number identified a significant relationship between *Sly*, *Ssty1*, *Srsy*, and *Rbmy* CNV, supporting a role for X-Y intragenomic conflict during spermatogenesis among B6-ChrY *M. m. domesticus* consomic strains.

A paternally transmitted POO effect on EAE susceptibility in female offspring was first identified among F_2_ intercross progeny generated from EAE-susceptible SJL/J (S) and EAE-resistant B10.S/SgMcdJ (B) mice [49]. Central nervous system infiltration and damage were only found to be different in female mice from the BS × BS intercross, whose grandsires and sires possessed the SJL/J ChrY. Female offspring of B6-ChrY consomic strains of mice exhibited a continuous distribution in EAE severity across the strains, consistent with quantitative inheritance. Analyses between EAE cumulative disease score with sex ratio and sperm head abnormalities identified a significant relationship, thus providing support for X-Y intragenomic conflict as the underlying factor driving the paternal POO effect on EAE among female offspring of B6-ChrY consomic mice [11]. Whether X-Y intragenomic conflict is the result of CNV in *Sly* and *Ssty1*, which are the ascribed mediators of X-Y intragenomic conflict [93,94], or whether other ChrY multicopy genes contributes to this phenomenon, remains to be determined.

The mammalian sex chromosome interaction is reminiscent of interactions between the ChrX and ChrY-encoded multi-copy genes *Stellate (Ste)* and *Suppressor of Stellate (Su(ste))* in *Drosophila*. Deletions of the *Drosophila* Y-linked *Su(ste)* locus cause spermatogenetic phenotypes, and it has been suggested that interactions between *Ste* and *Su(ste)* during spermatogenesis might influence the sex-ratio through the differential viability of X-bearing and Y-bearing gametes [103,104]. However, the mechanisms through which *Su(ste)* suppresses the *Ste* locus on ChrX remain poorly elucidated. Similarly, the mechanism by which ChrY CNV impacts the paternal POO effect on EAE in female offspring remains unknown.

Whether X-Y intragenomic conflict occurs in humans remains unknown. Nonetheless, families of probands with female-biased sexual dimorphism in autoimmune disease prevalence (multiple sclerosis, systemic lupus erythematosus, rheumatoid arthritis, and pauciarticular onset juvenile rheumatoid arthritis) exhibit a female-biased sex-ratio, whereas families of probands affected with non-sexually dimorphic autoimmune diseases (systemic onset juvenile rheumatoid arthritis and type 1 diabetes) exhibit unbiased sex-ratios, suggests that X-Y intragenomic conflict may play a role in autoimmune disease susceptibility in humans [11]. Furthermore, as in *Drosophila* and mice, the human ChrY contains over 10 Mb of ampliconic sequence containing multicopy gene families critical for spermatogenesis, some of which are amplified versions of ChrX homologs with testis-specific expression patterns, that represent potential candidates for X-Y intragenomic conflict [105].

## Conclusions

Differences between male and female physiology and behavior are more often the rule than the exception, and experimental animal models have contributed greatly to our current understanding of adult sexual dimorphisms as a function of sex hormones and sex Chrs. It is well established that ChrY protein-coding genes are critical for the sexual development and fertility of males. ChrY also has a long-standing history in regulating differences in brain and behavior between the sexes [25]. The contribution of ChrY to phenotype diversity beyond those traditionally ascribed is rapidly growing, yet insight into the mechanistic basis whereby ChrY polymorphism mediates genome-wide gene expression and sex differences is still speculative. While differential gene expression of ChrY-encoded genes, such as *Sry* in the brain, may contribute to ChrY’s gene regulatory capacity [106], this cannot explain the phenotypic differences among tissues lacking detectable expression of ChrY-encoded genes. Of particular interest will be to further explore the contribution of CNV between the homologous X and Y multicopy genes on genome-wide gene regulation and disease susceptibility in mice [60], which has also been proposed to contribute to sex differences in physiology and disease among other mammals, including humans [83]. In fact, a similar genetic mechanism has been established for ChrY of *Drosphila* subspecies, supporting the existence of an evolutionarily conserved mechanism of gene regulation by ChrY [76]. Future investigations into the genetic and molecular mechanisms that establish ChrY as a member of the regulatory genome in males, and as a factor influencing paternal POO effects in female offspring, will be aided by the sequencing efforts put forth for mammalian ChrYs [107-109], especially mouse [10].

## Abbreviations

Chr: Chromosome
ChrY: Y chromosome
ChrX: X chromosome
CNV: Copy number variation
GDX: Gonadectomized
FCG: Four core genotypes
B6: C57BL/6 J
EAE: Experimental allergic encephalomyelitis
QTL: Quantitative trait locus
eQTL: Expression quantitative trait locus
POO: Parent-of-origin
